# Supplementary figures and images for: ATG conjugation–dependent/independent mechanisms underlie lysosomal stress–induced TFEB regulation
Source: J Cell Biol. 2025 Aug 29;224(10):e202307079. doi: 10.1083/jcb.202307079 (PMC12396377; doi:10.1083/jcb.202307079)

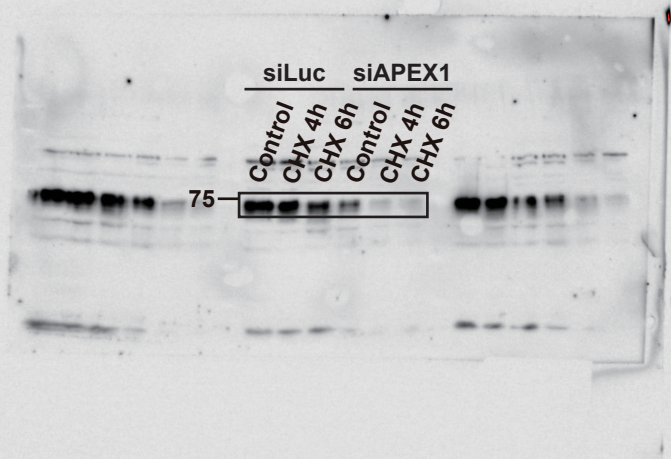

TFEB

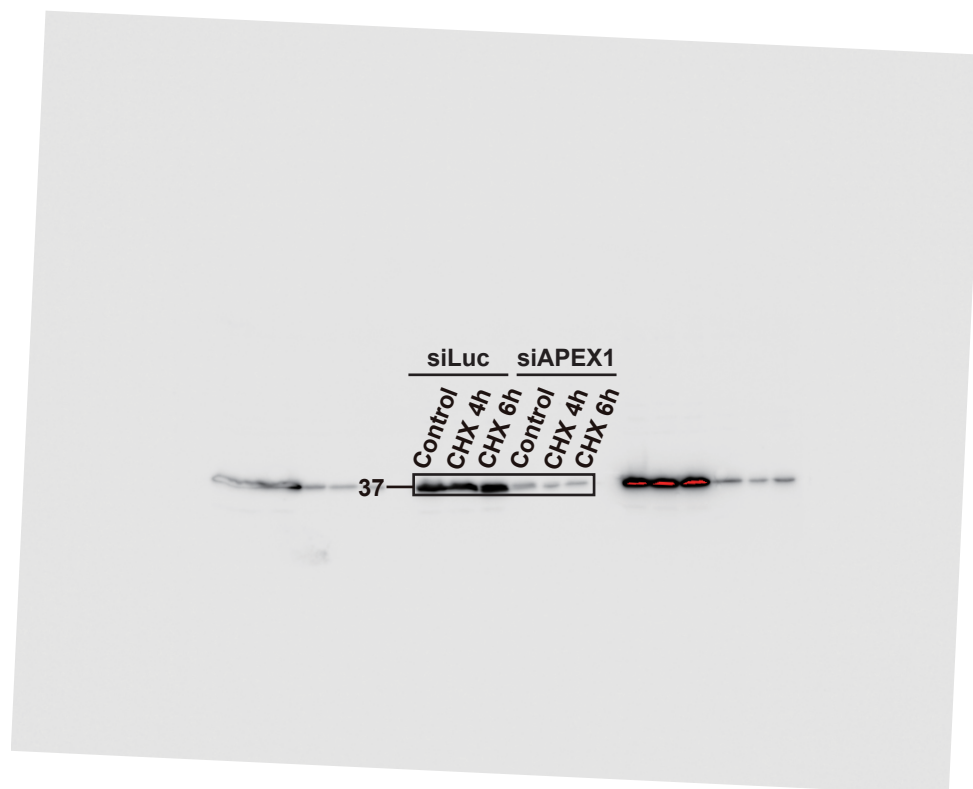

APEX1

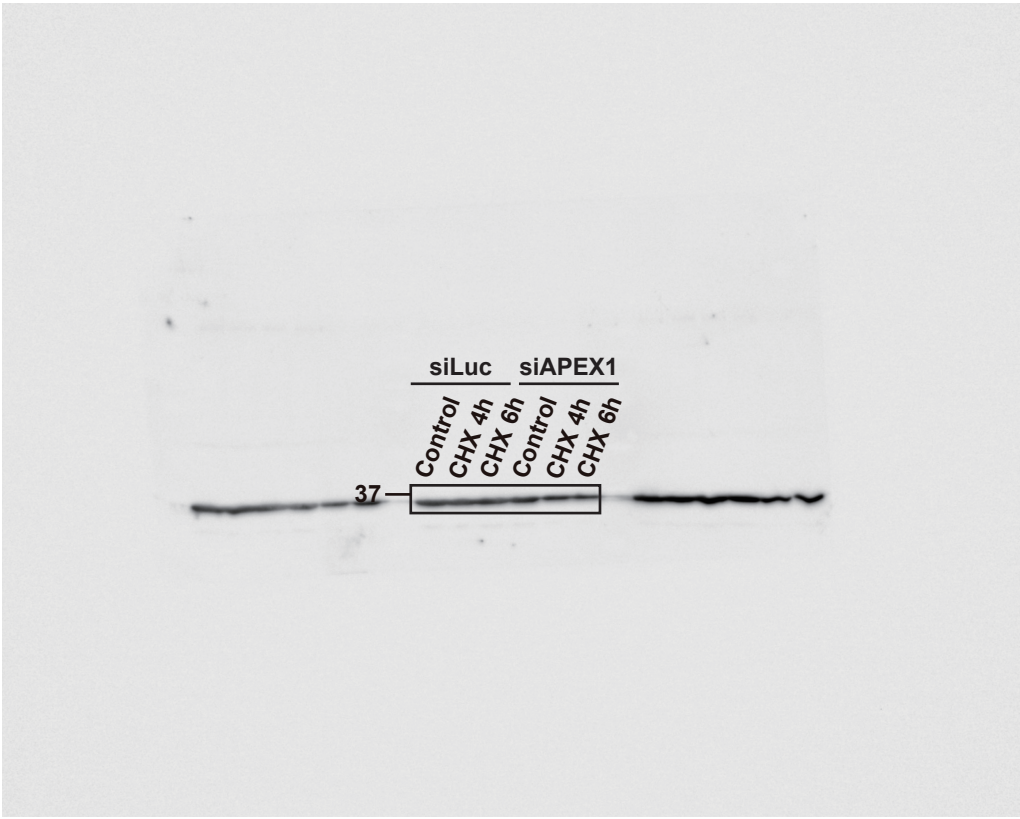

GAPDH

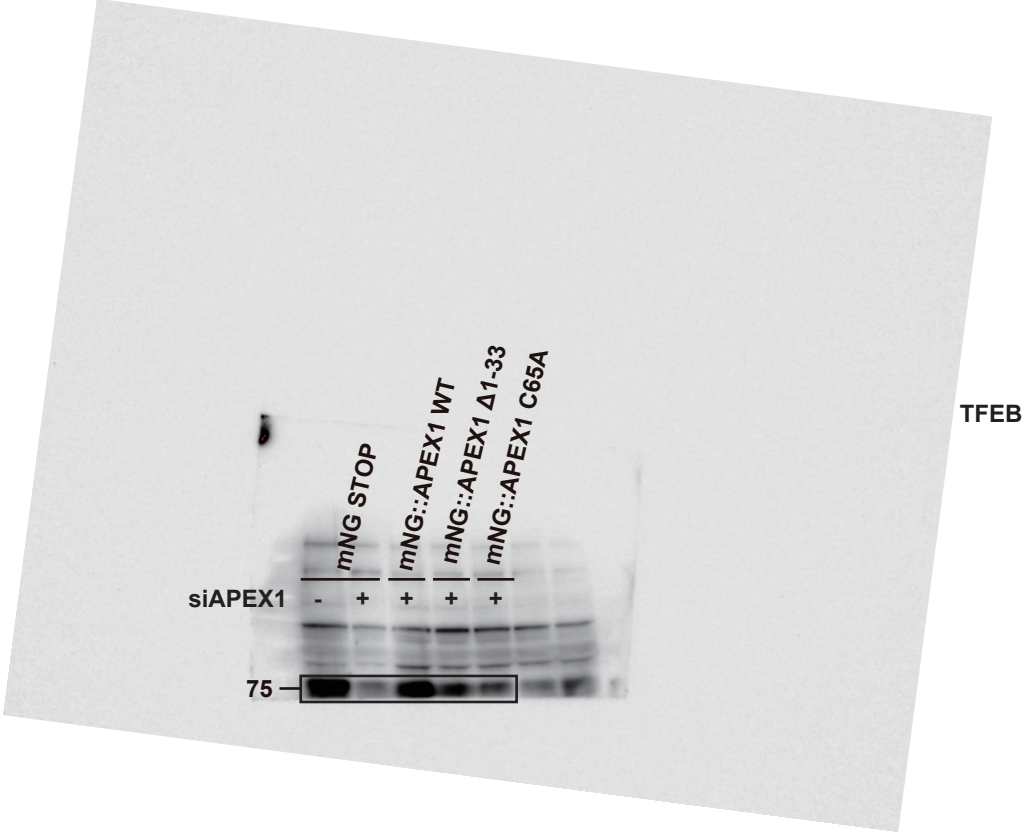

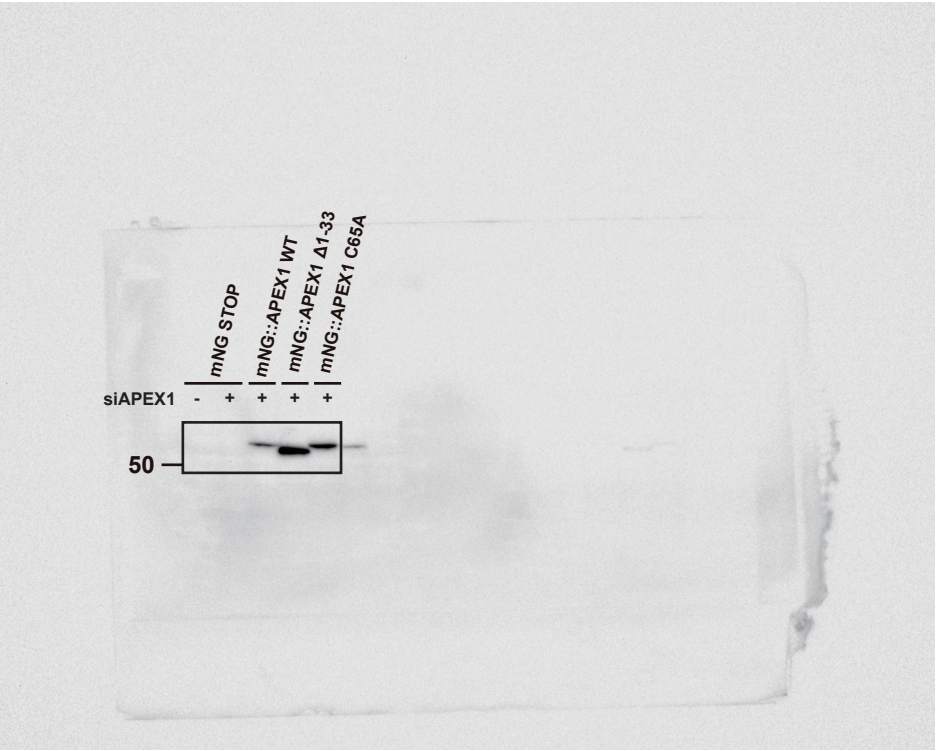

mNG

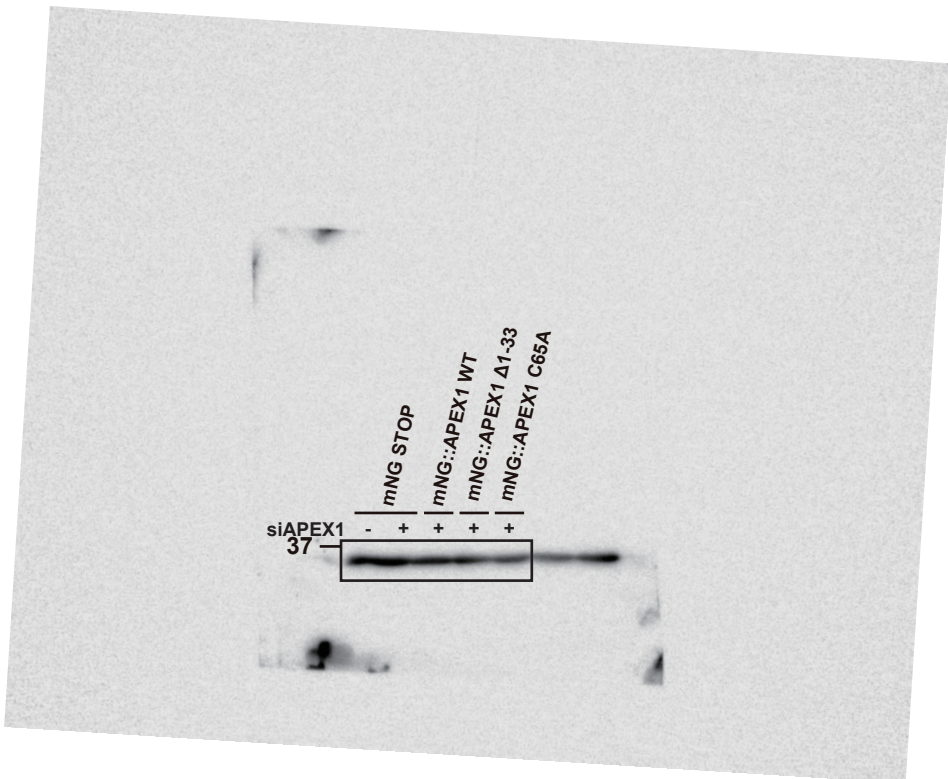

GAPDH

Supplement: SourceData F2 — is the source file for Fig. 2. [file jcb_202307079_sourcedataf2.pdf]

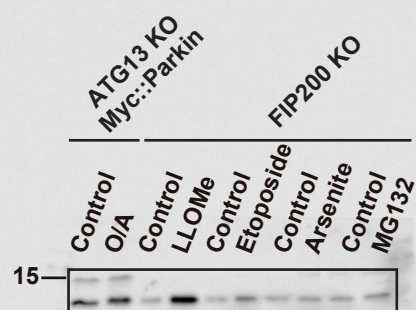

LC3

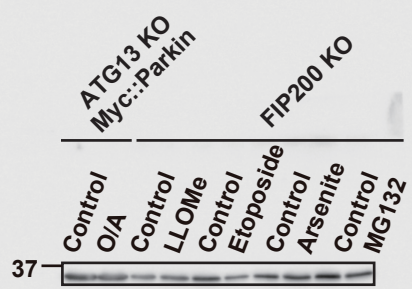

GAPDH

Supplement: SourceData F4 — is the source file for Fig. 4. [file jcb_202307079_sourcedataf4.pdf]

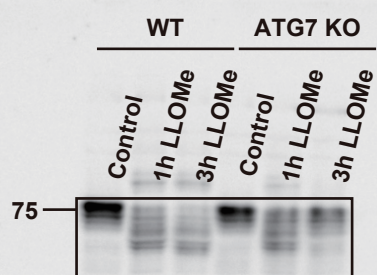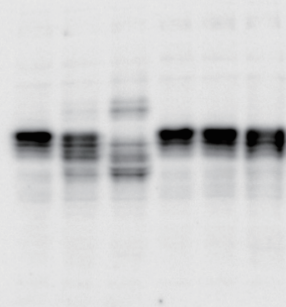

TFEB

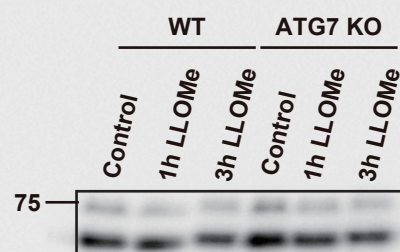

S6K

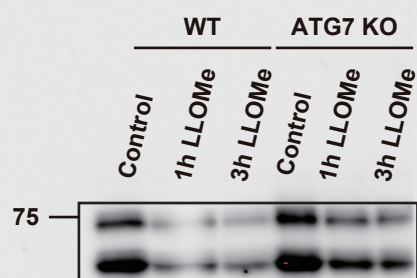

p-S6K

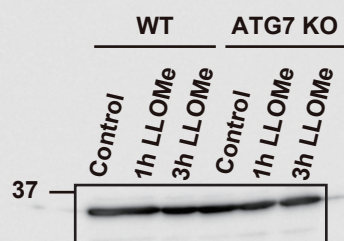

GAPDH

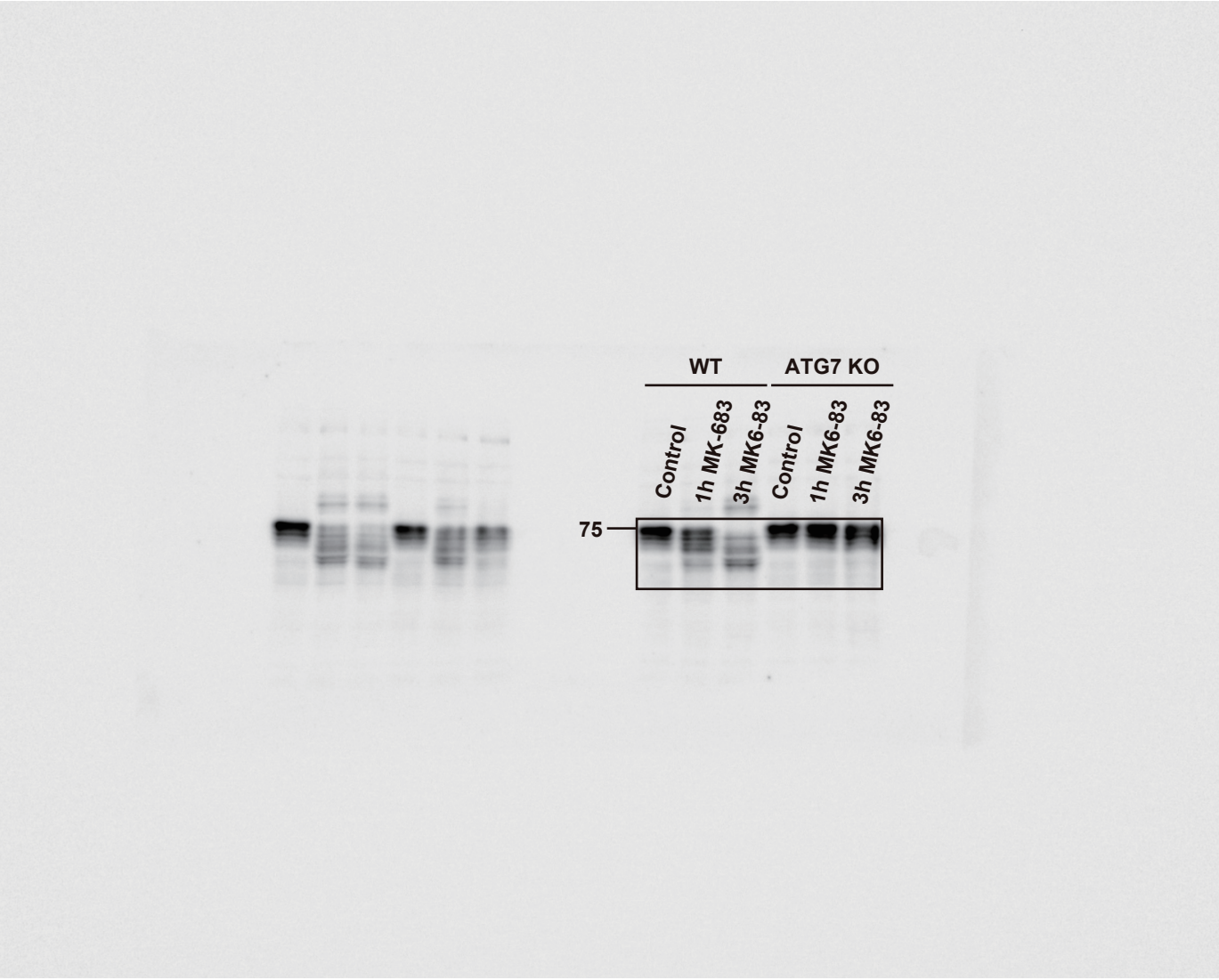

TFEB

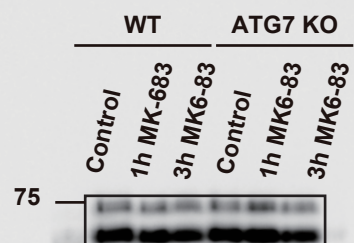

S6K

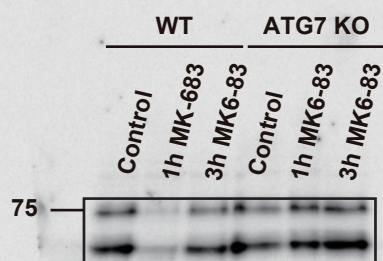

p-S6K

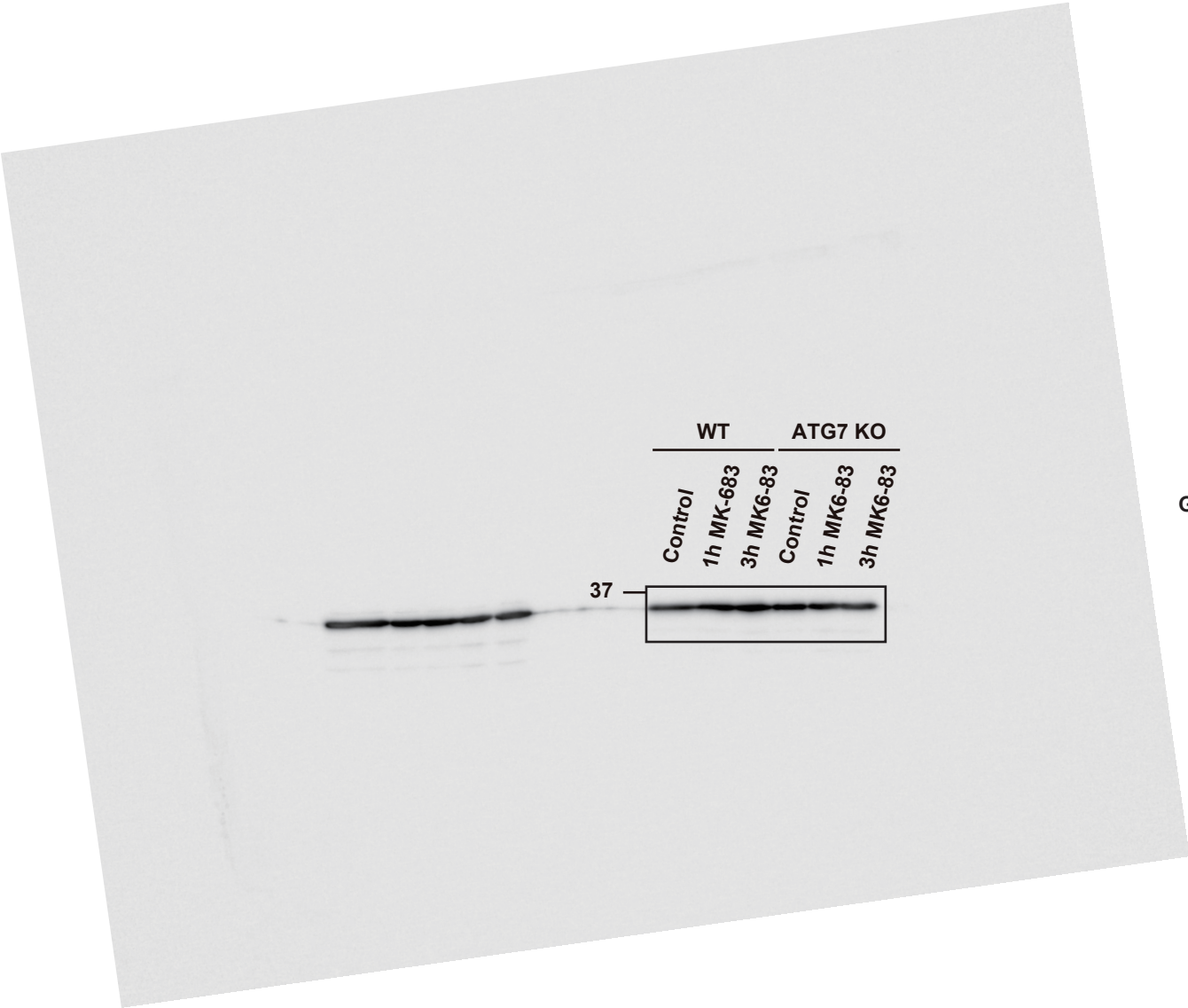

GAPDH

Supplement: SourceData FS1 — is the source file for Fig. S1. [file jcb_202307079_sourcedatafs1.pdf]

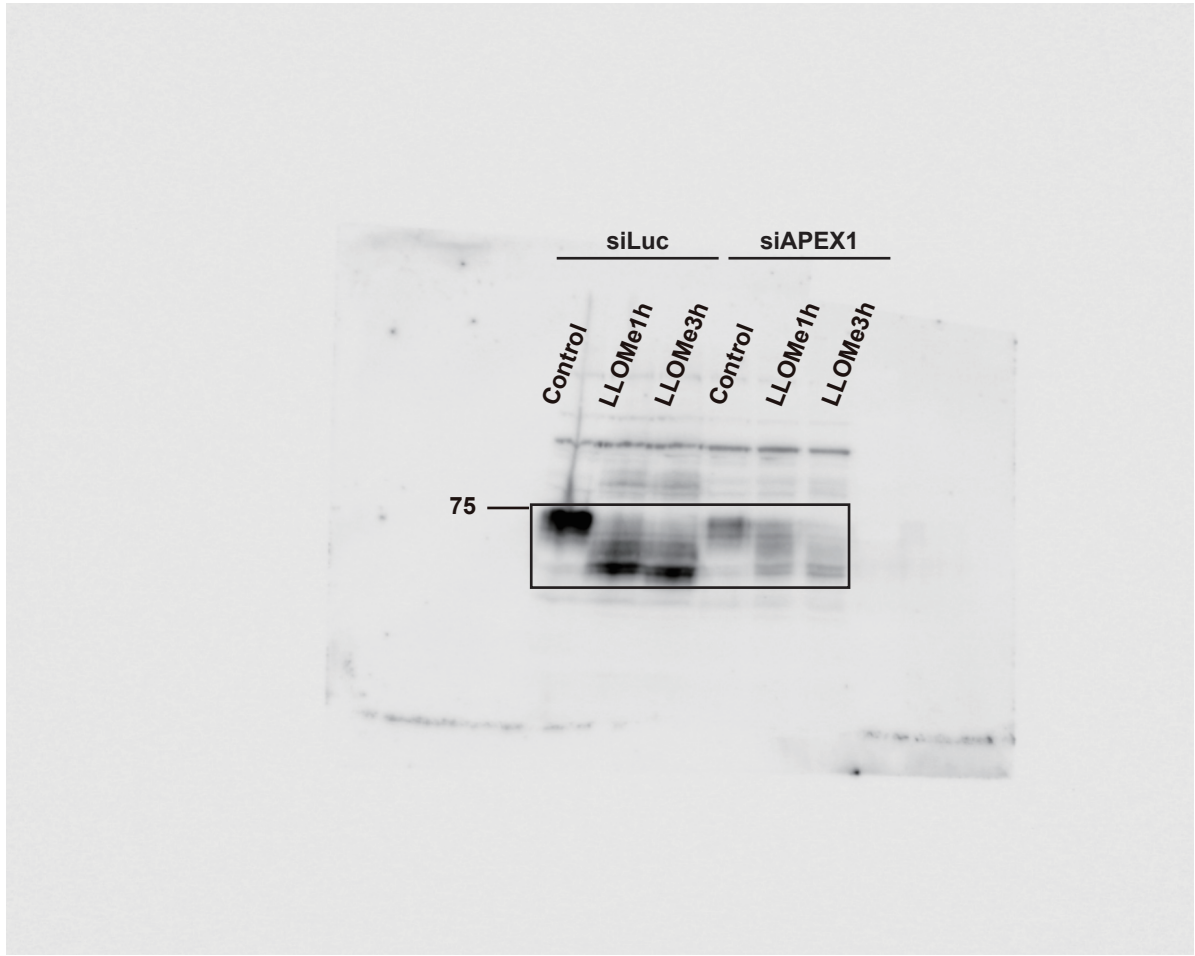

TFEB

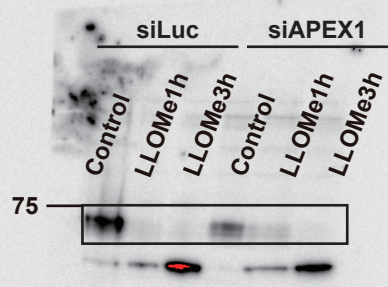

p-TFEB  
(Ser211)

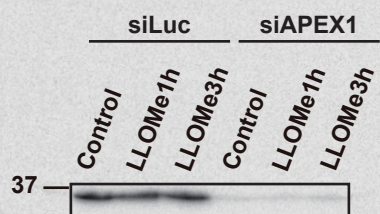

APEX1

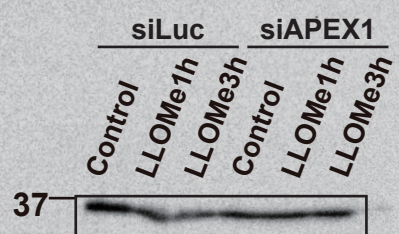

GAPDH

Supplement: SourceData FS3 — is the source file for Fig. S3. [file jcb_202307079_sourcedatafs3.pdf]

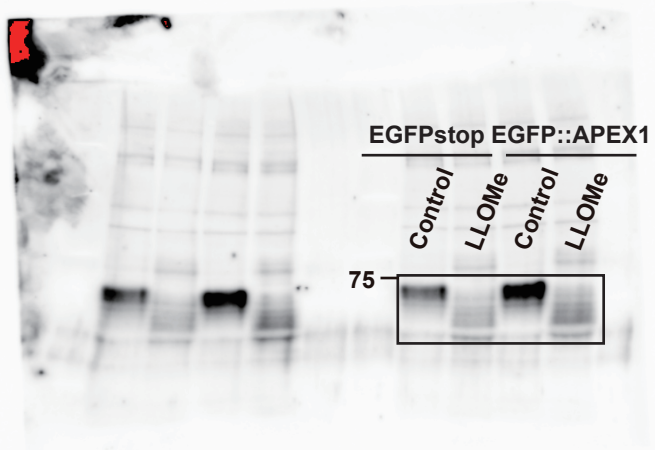

TFEB

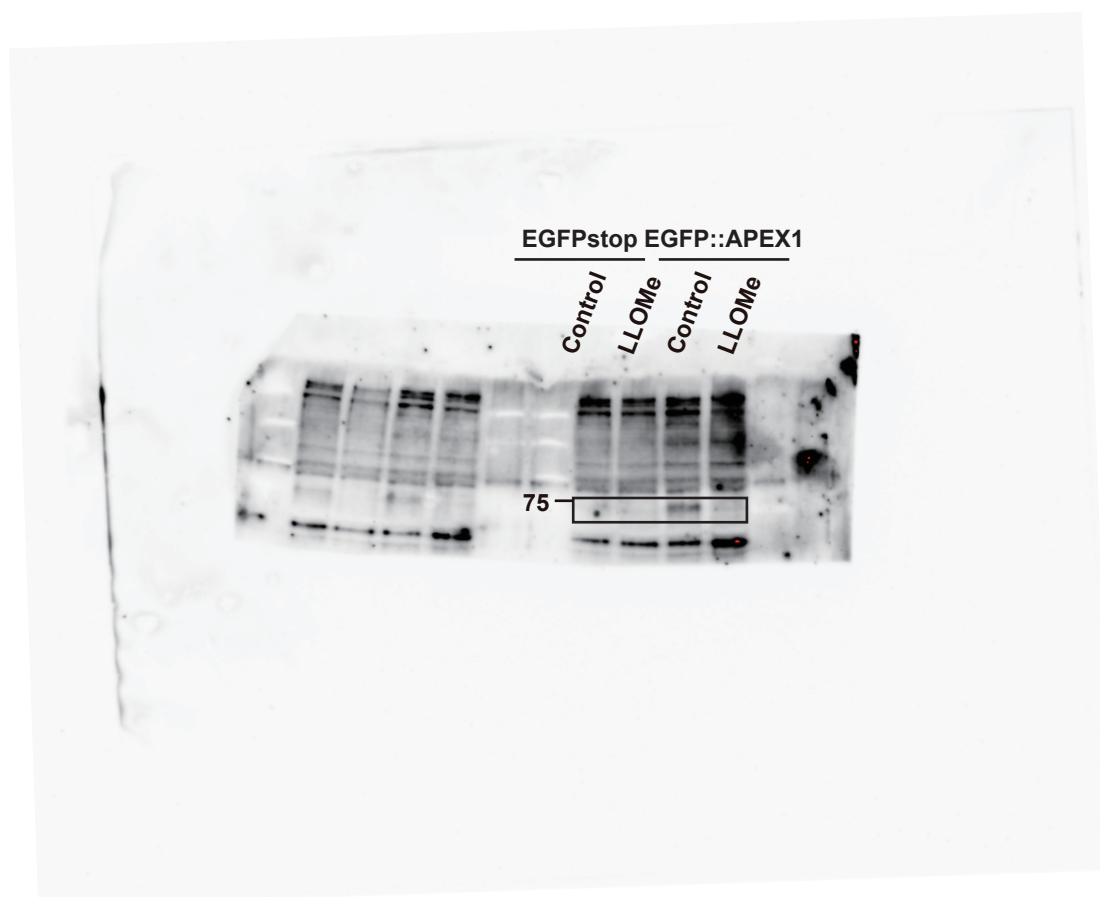

p-TFEB(Ser211)

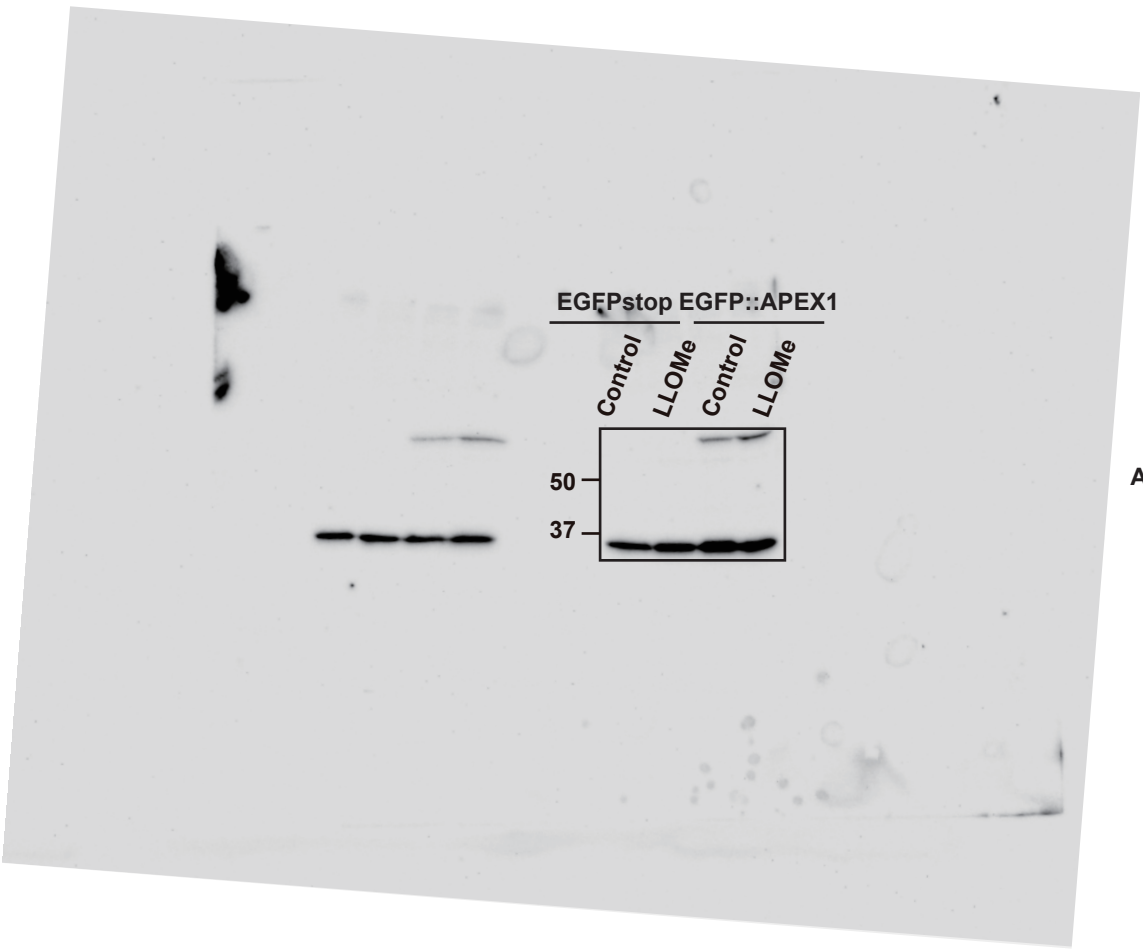

APEX1

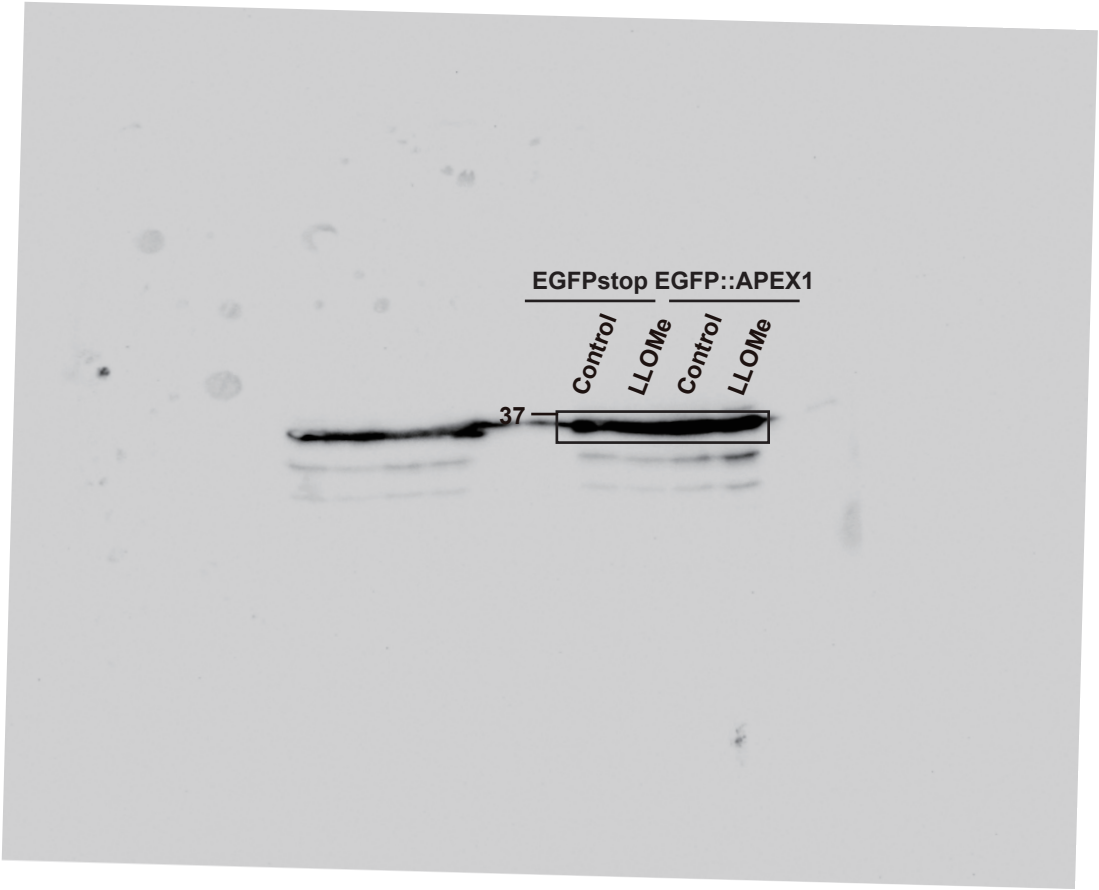

GAPDH

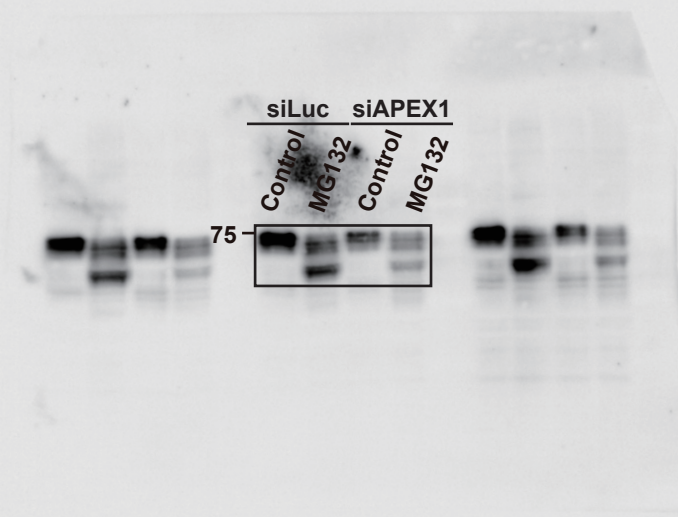

TFEB

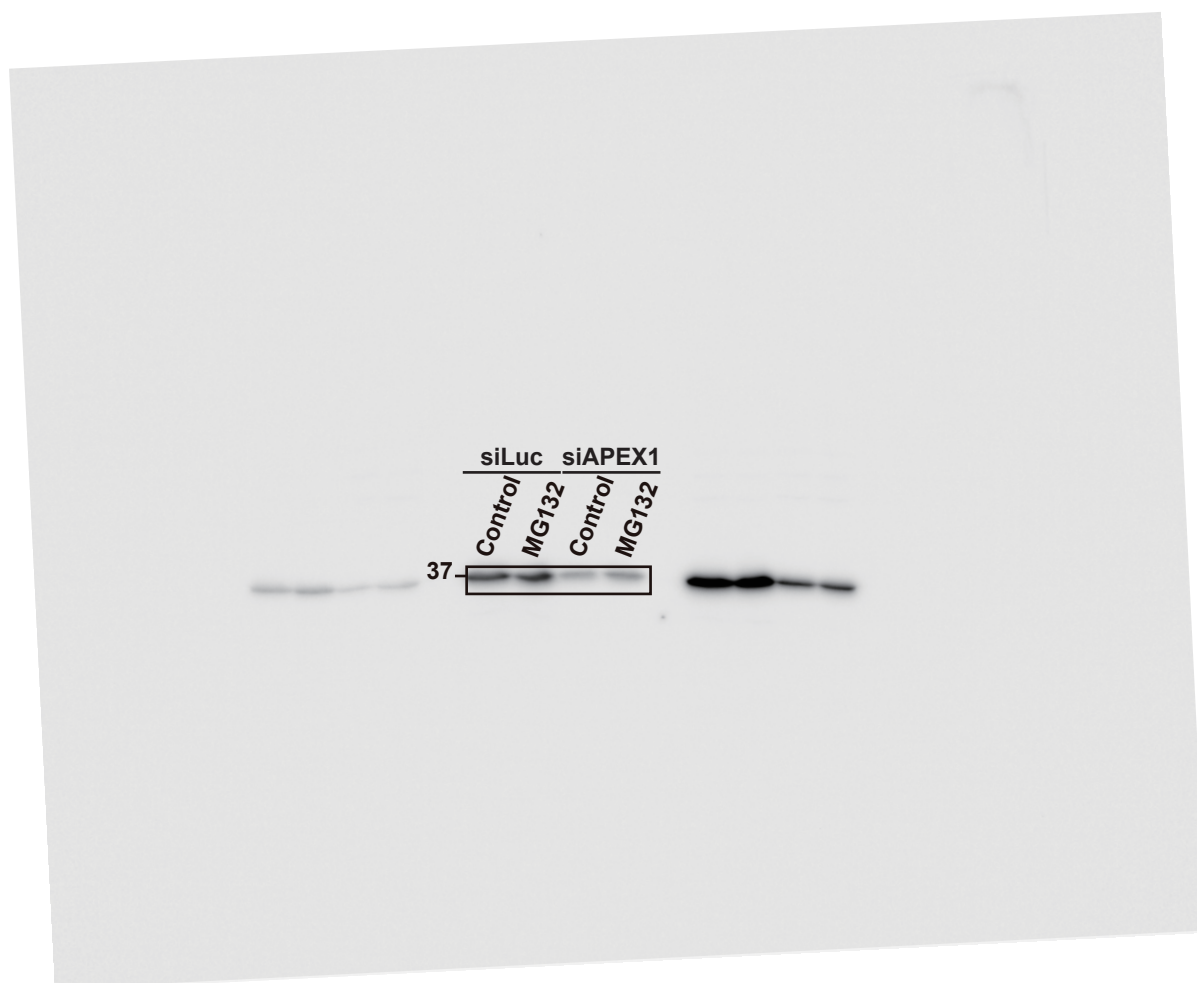

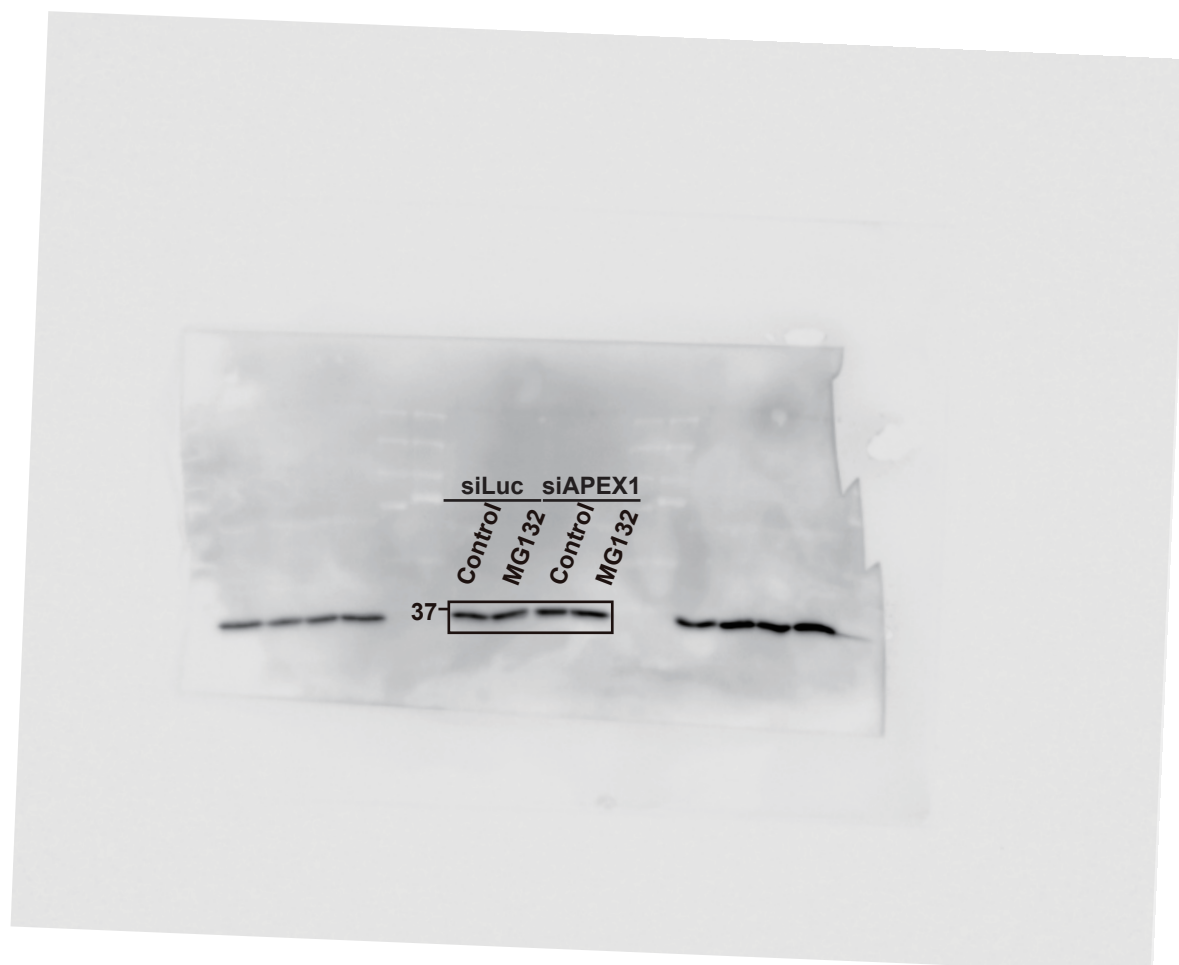

GAPDH

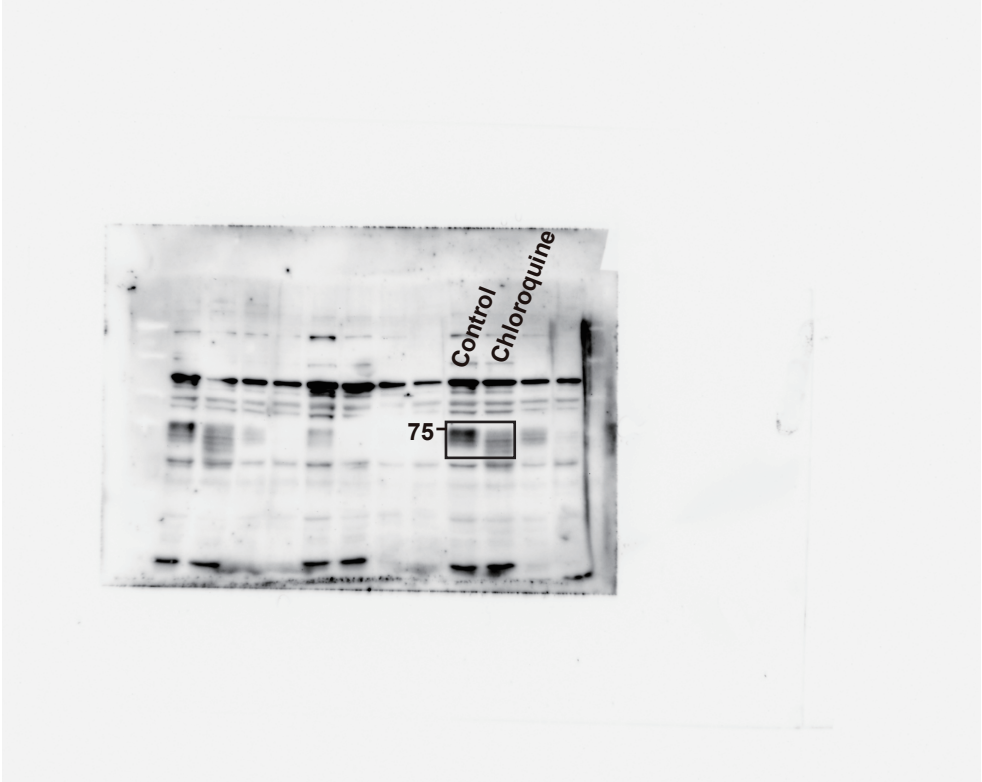

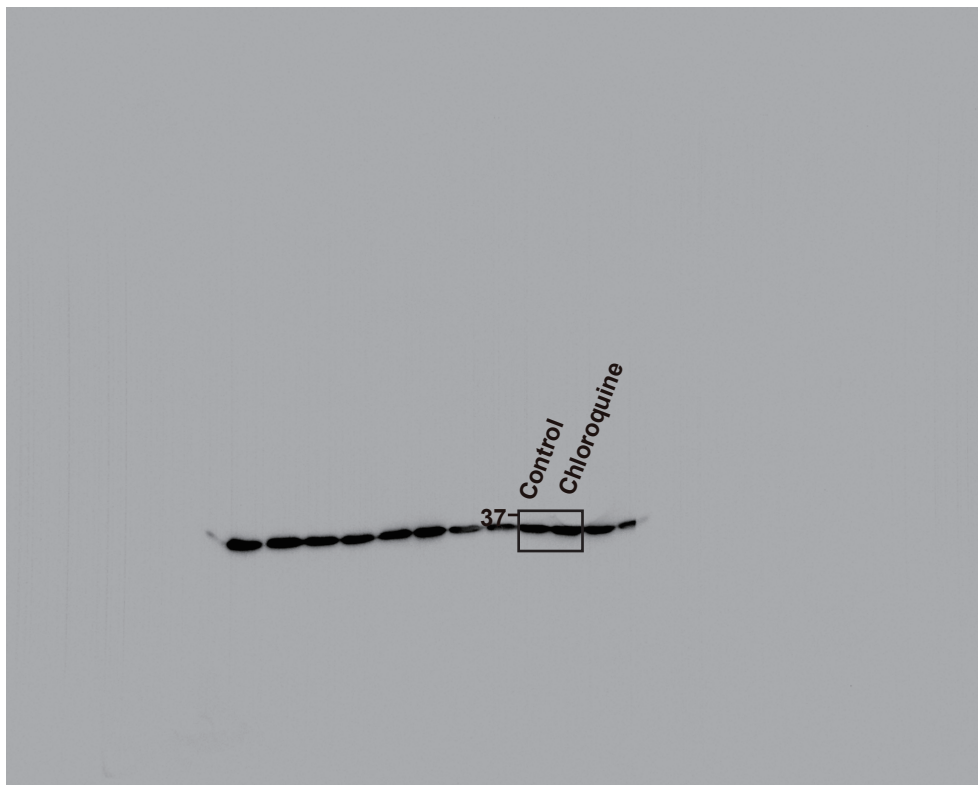

GAPDH

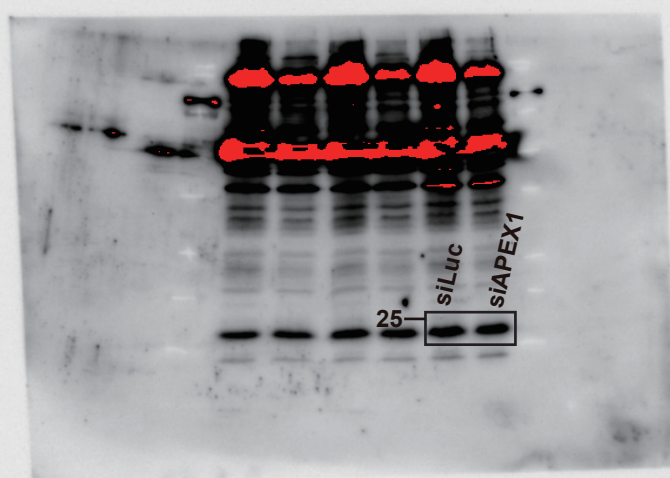

$\gamma$ H2AX

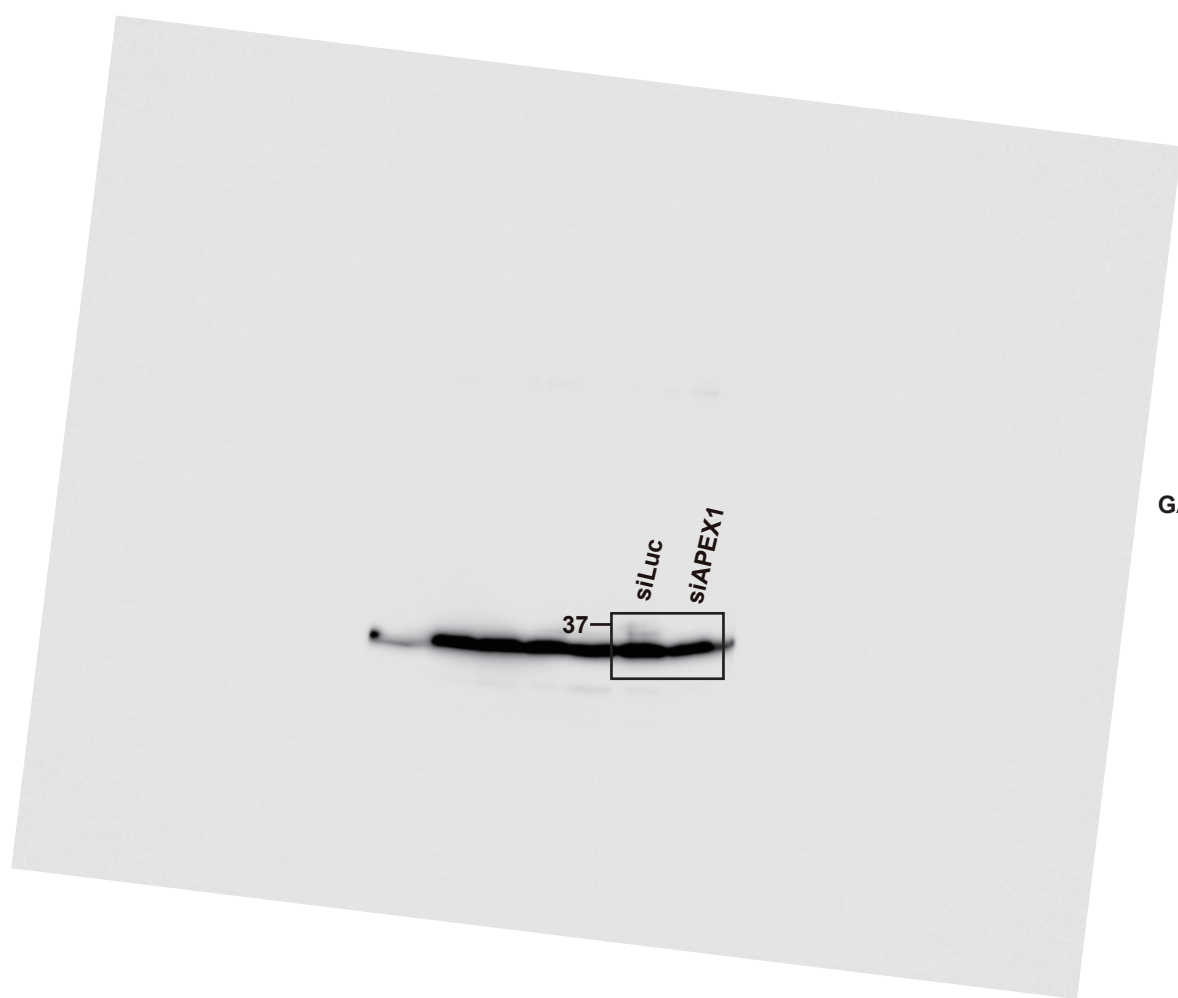

GAPDH

37

siLuc

siAPEX1

Supplement: SourceData FS4 — is the source file for Fig. S4. [file jcb_202307079_sourcedatafs4.pdf]

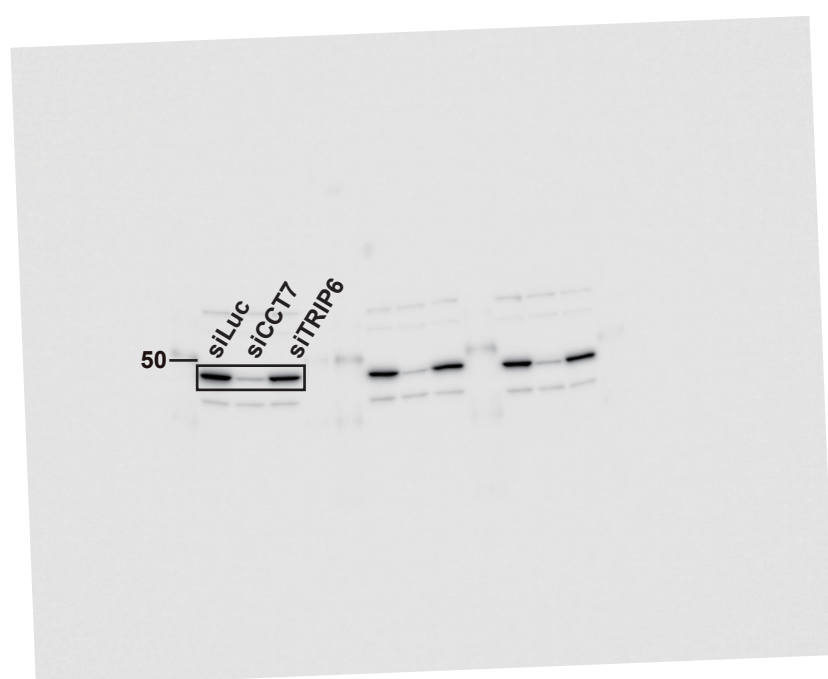

50  
siLuc  
siCCT7  
siTRIP6

TRIP6

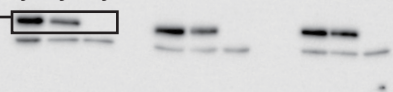

37— siLuc  
siCCT7  
siTRIP6

GAPDH

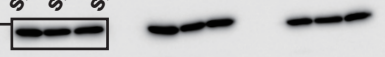

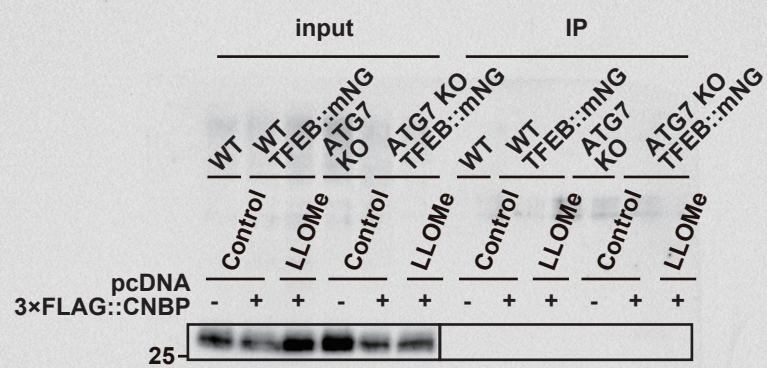

FLAG(FLAG::CNBP)

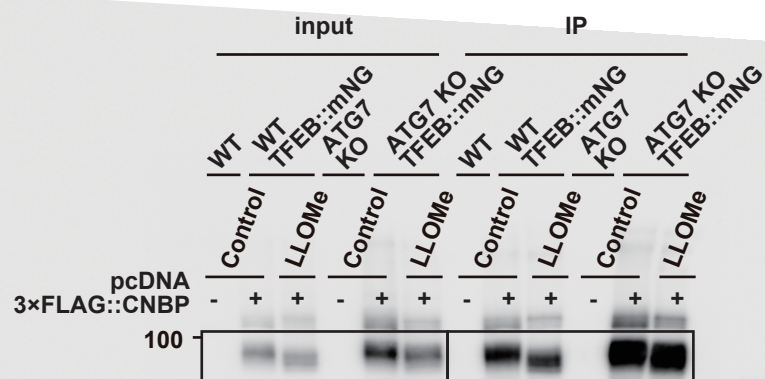

TFEB(TFEB::mNG)

Supplement: SourceData FS5 — is the source file for Fig. S5. [file jcb_202307079_sourcedatafs5.pdf]

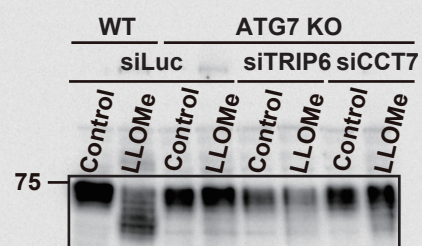

TFEB

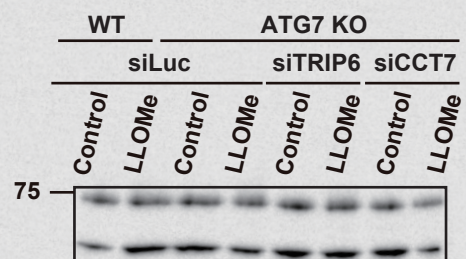

AMPK

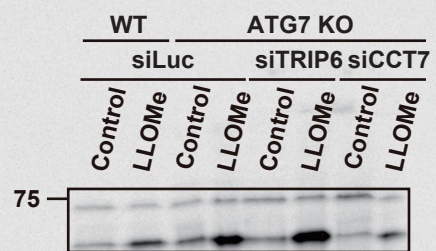

p-AMPK

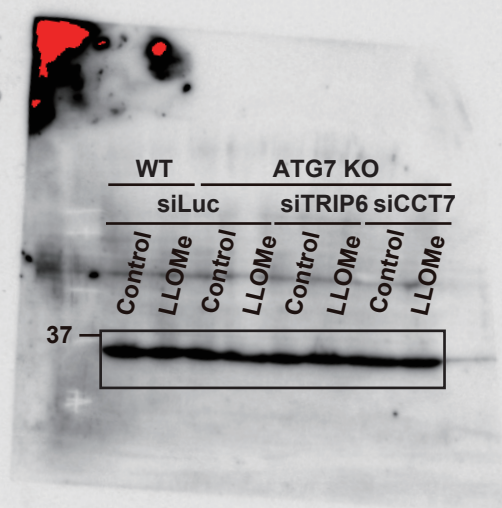

GAPDH

Supplement: SourceData FS6 — is the source file for Fig. S6. [file jcb_202307079_sourcedatafs6.pdf]

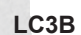

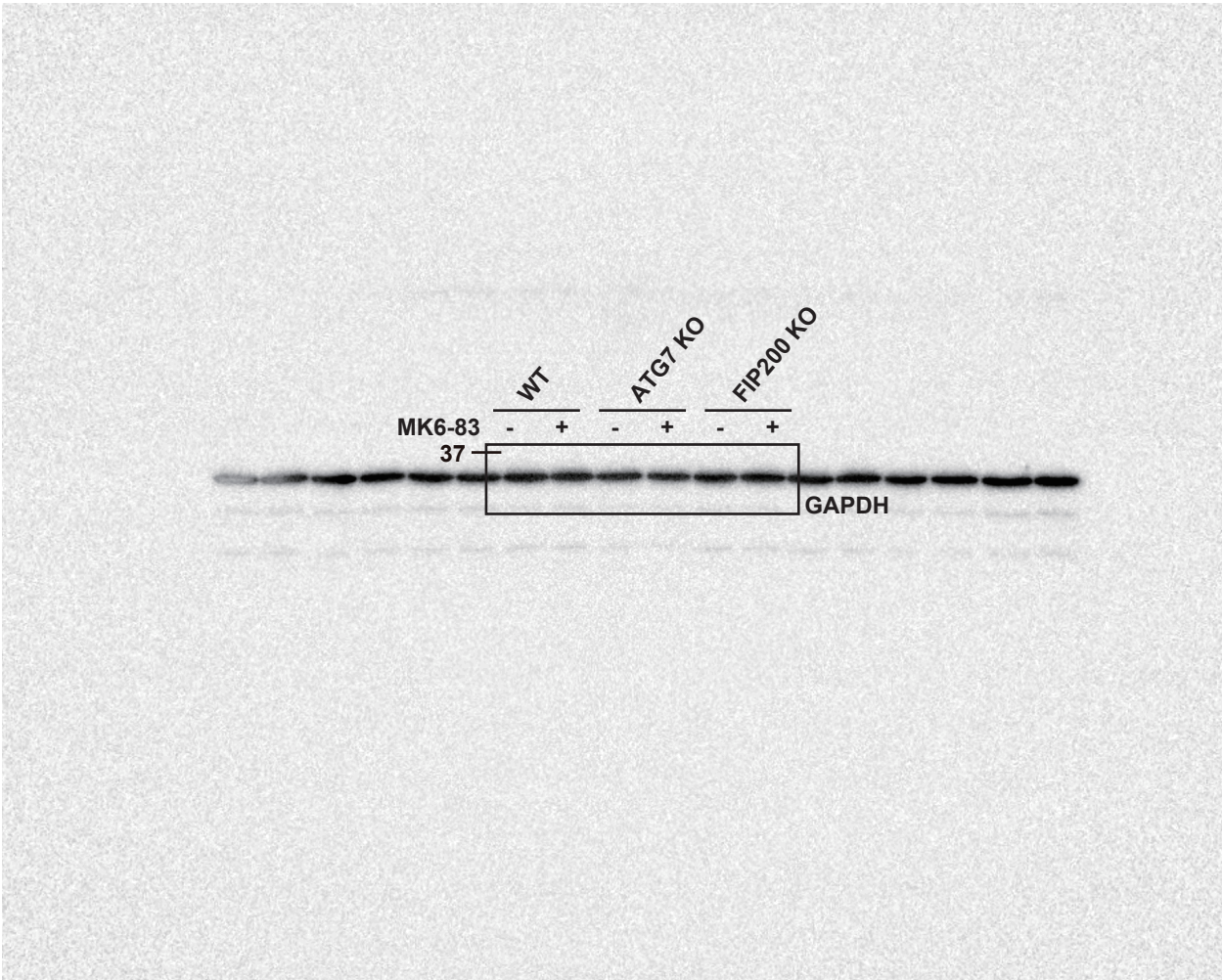

GAPDH

Supplement: SourceData FS7 — is the source file for Fig. S7. [file jcb_202307079_sourcedatafs7.pdf]
