# Supplementary material for: ATG conjugation–dependent/independent mechanisms underlie lysosomal stress–induced TFEB regulation
Source: J Cell Biol. 2025 Aug 29;224(10):e202307079. doi: 10.1083/jcb.202307079 (PMC12396377; doi:10.1083/jcb.202307079)
Supplement: SourceData F3 — is the source file for Fig. 3. [file jcb_202307079_sourcedataf3.pdf]

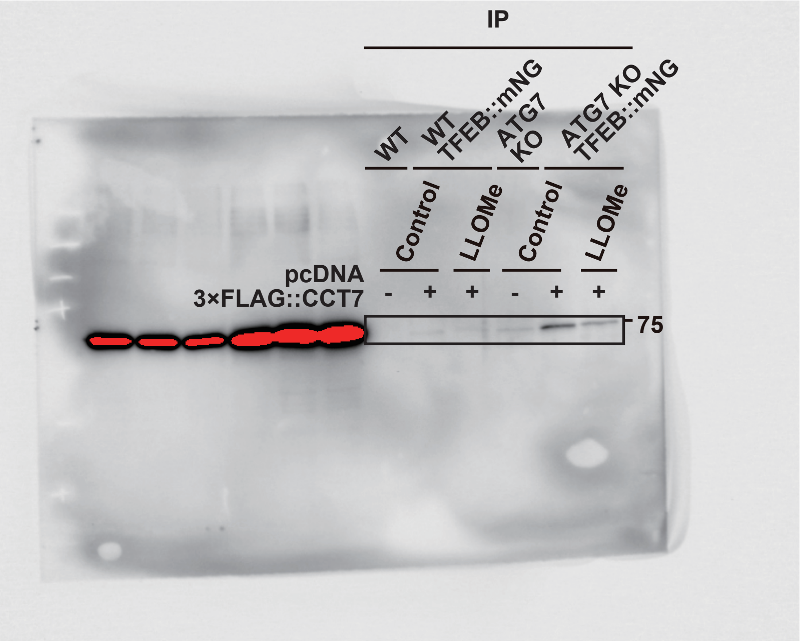

FLAG(FLAG::CCT7)

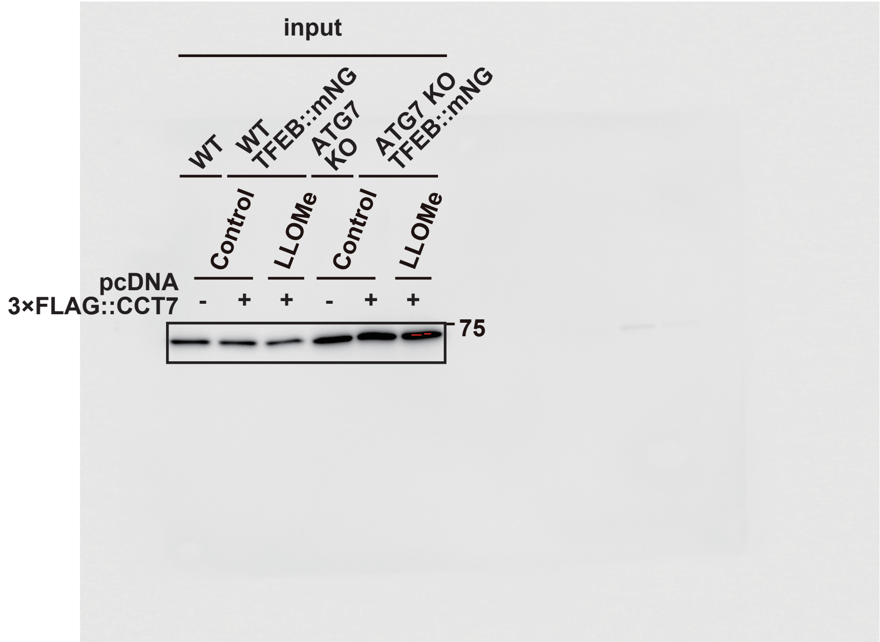

FLAG(FLAG::CCT7)

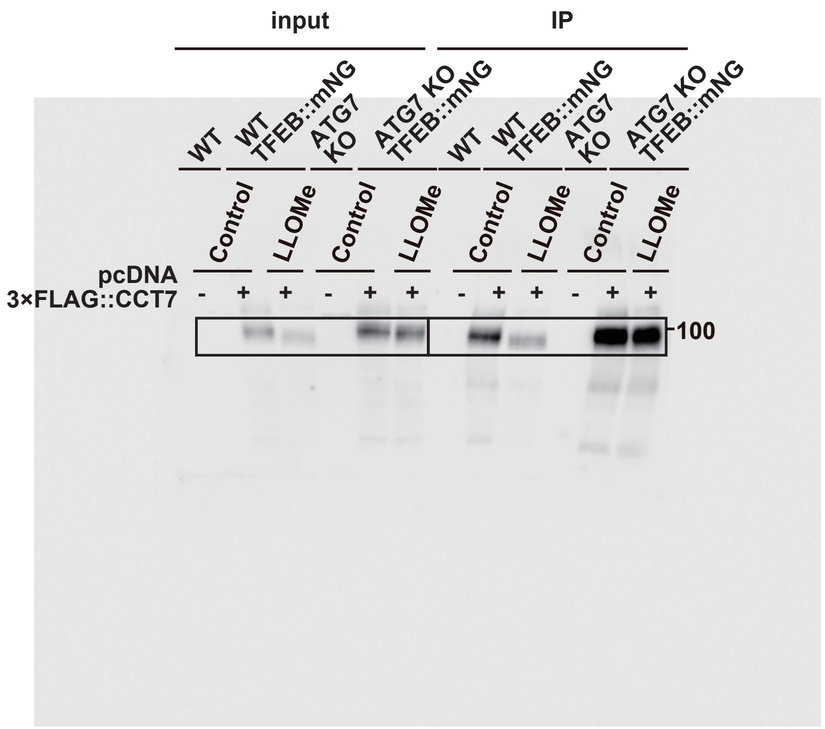

TFEB(TFEB::mNG)

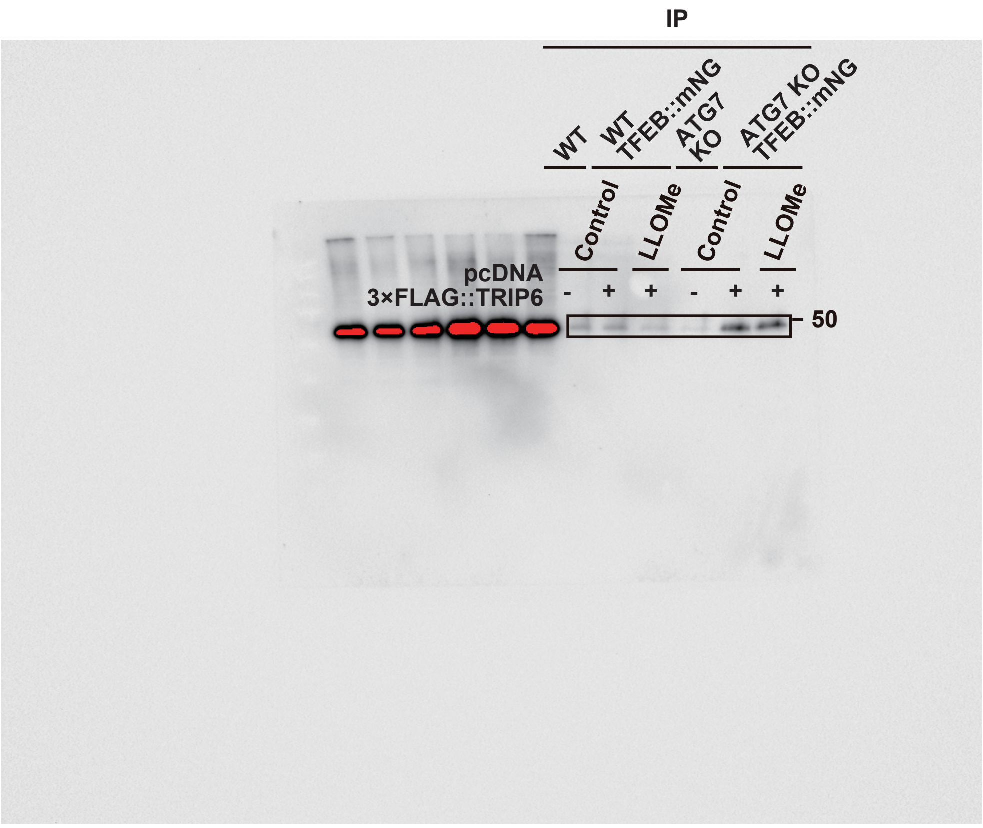

FLAG(FLAG::TRIP6)

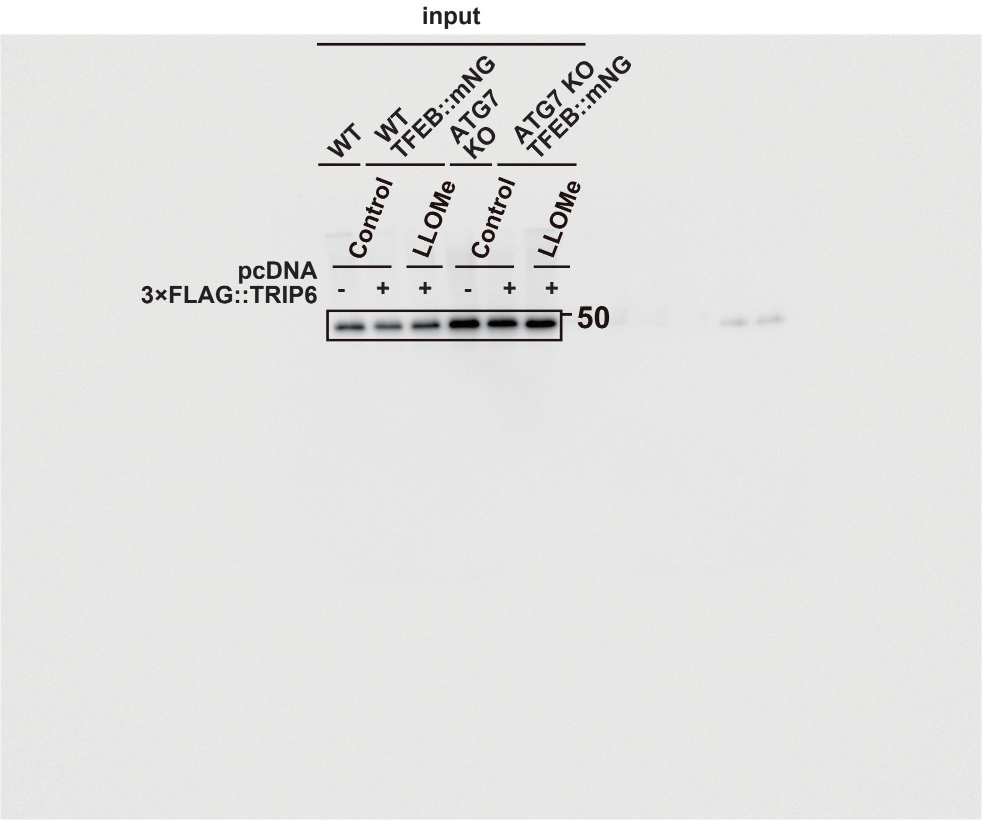

FLAG(FLAG::TRIP6)

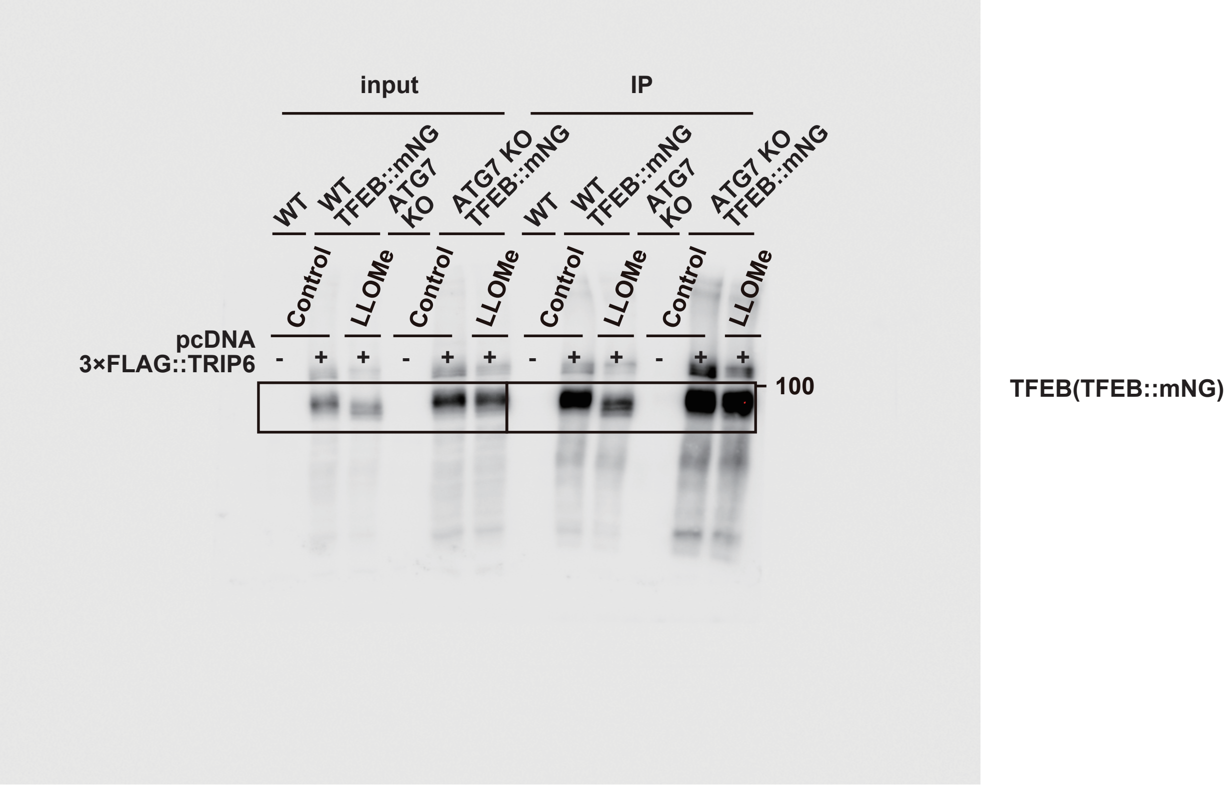
input
IP
WT
WT TFEB::mNG
ATG7 KO
ATG7 KO TFEB::mNG
Control
LLOMe
pcDNA
3×FLAG::TRIP6
- + + - + + - + + - + +
100
TFEB(TFEB::mNG)

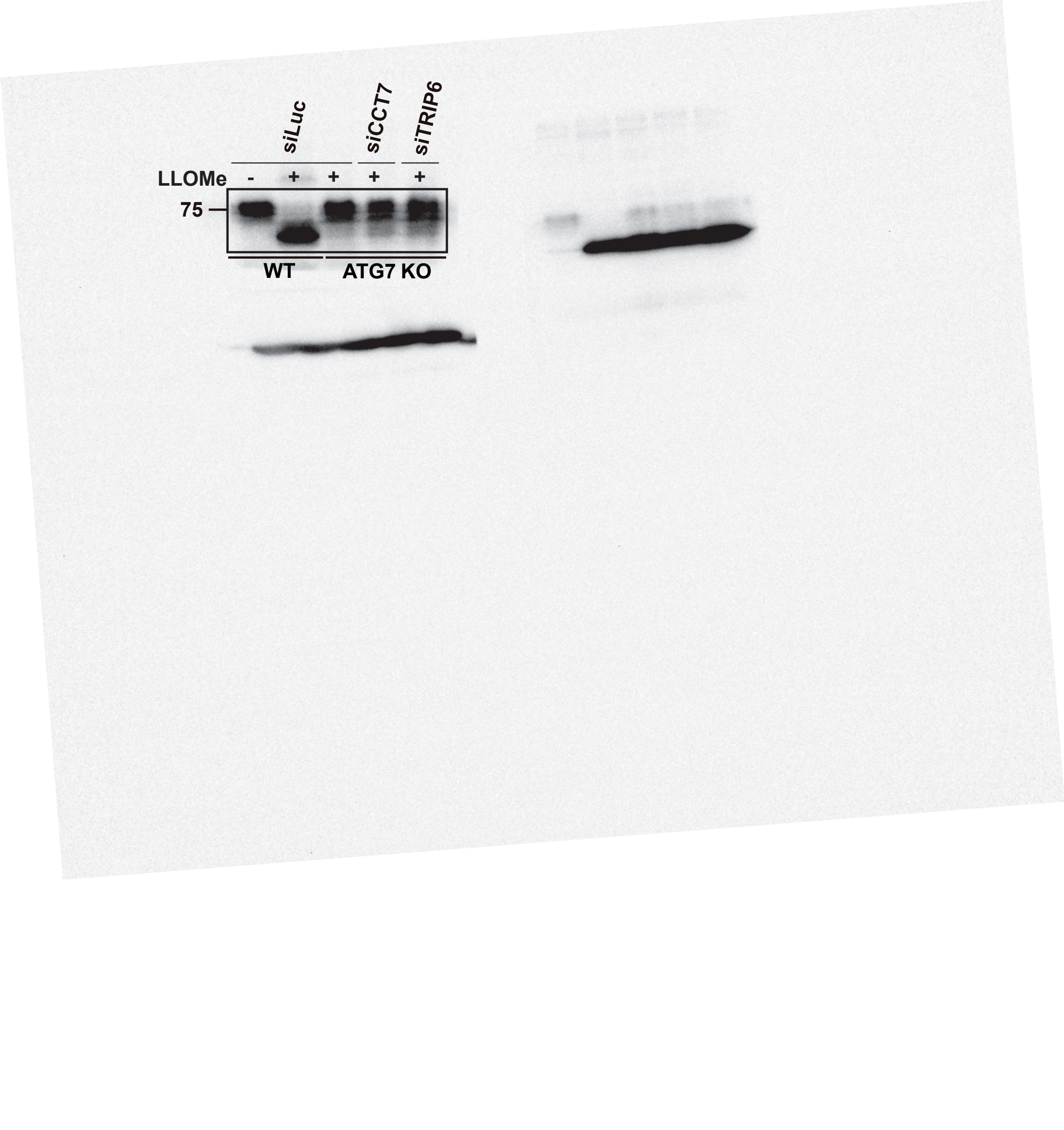

TFEB

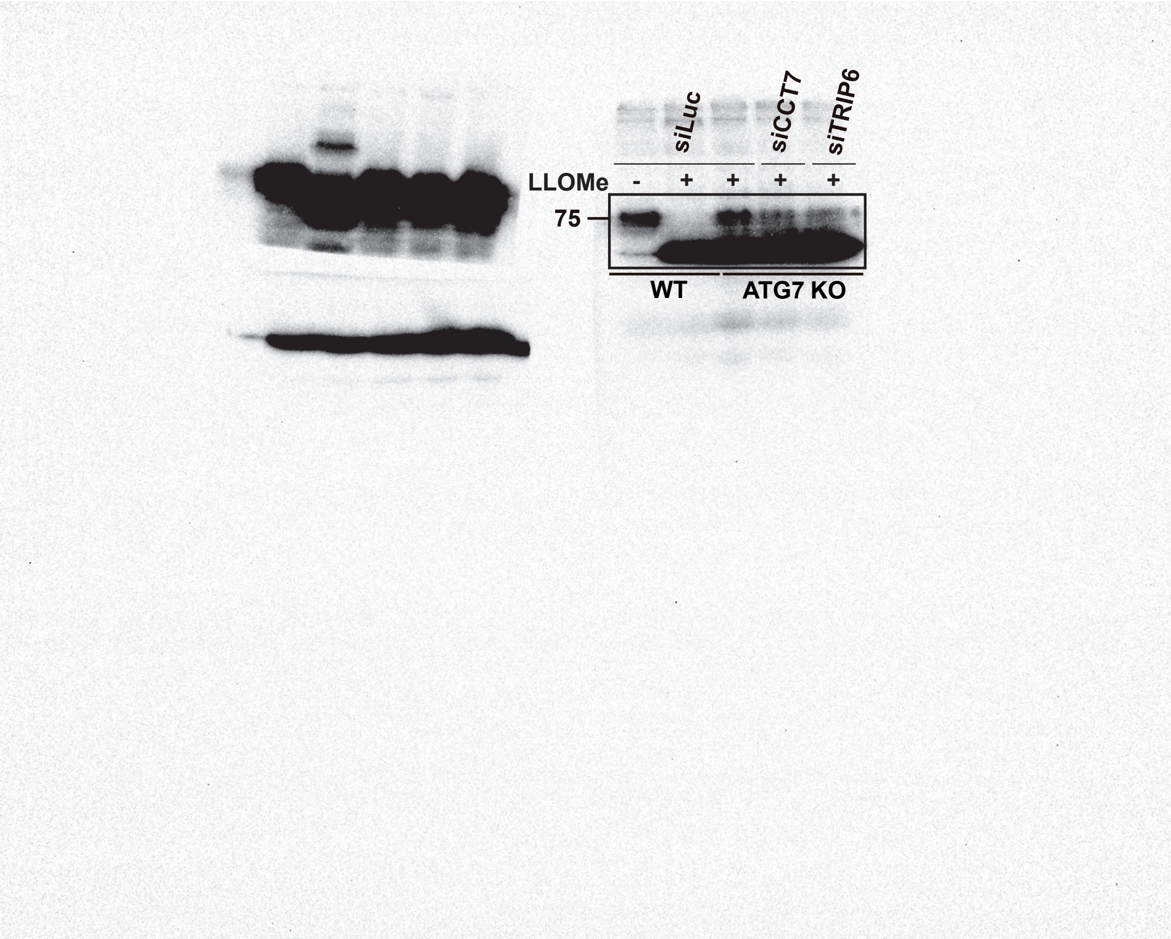

p-TFEB
(Ser211)

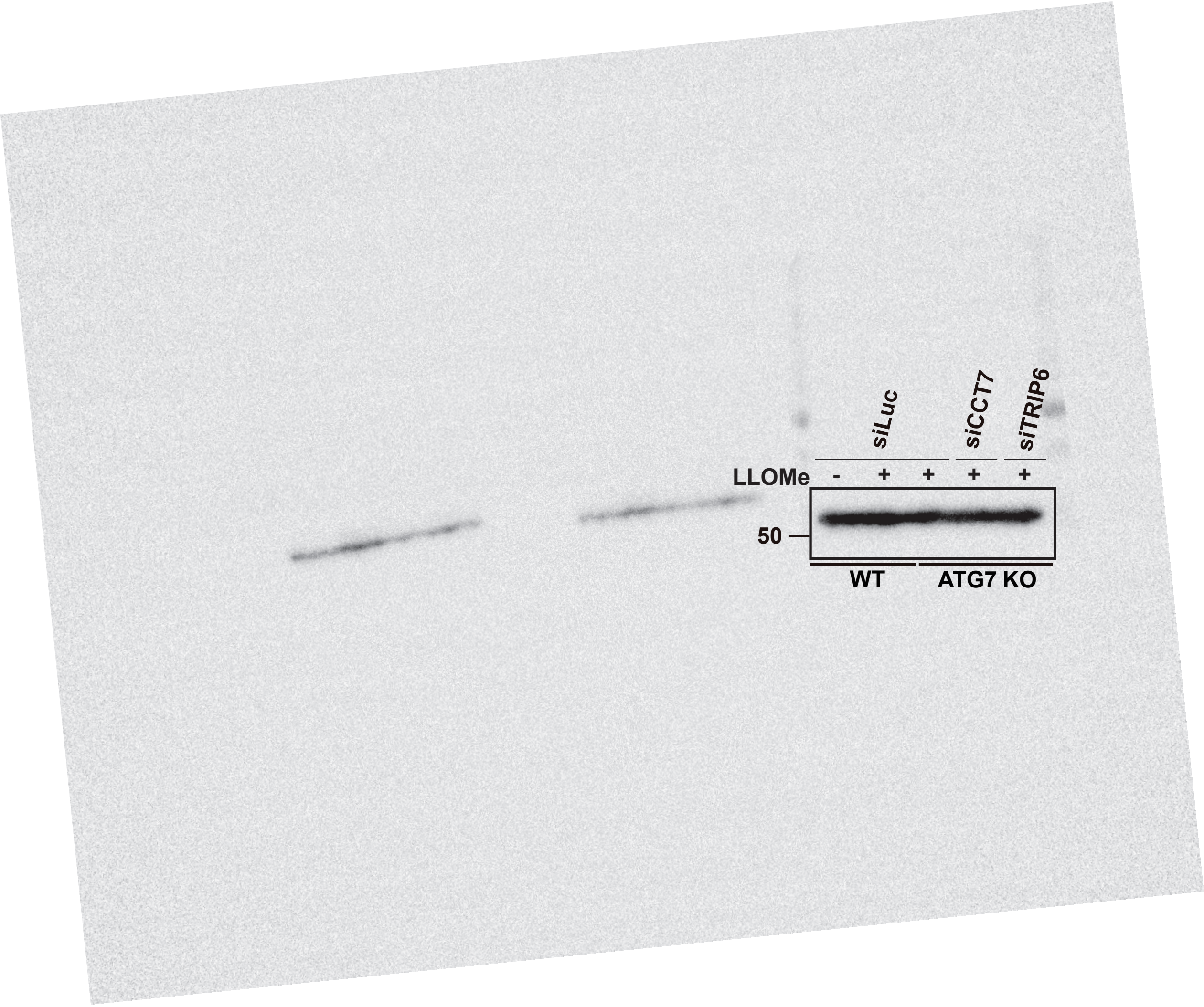

tubulin

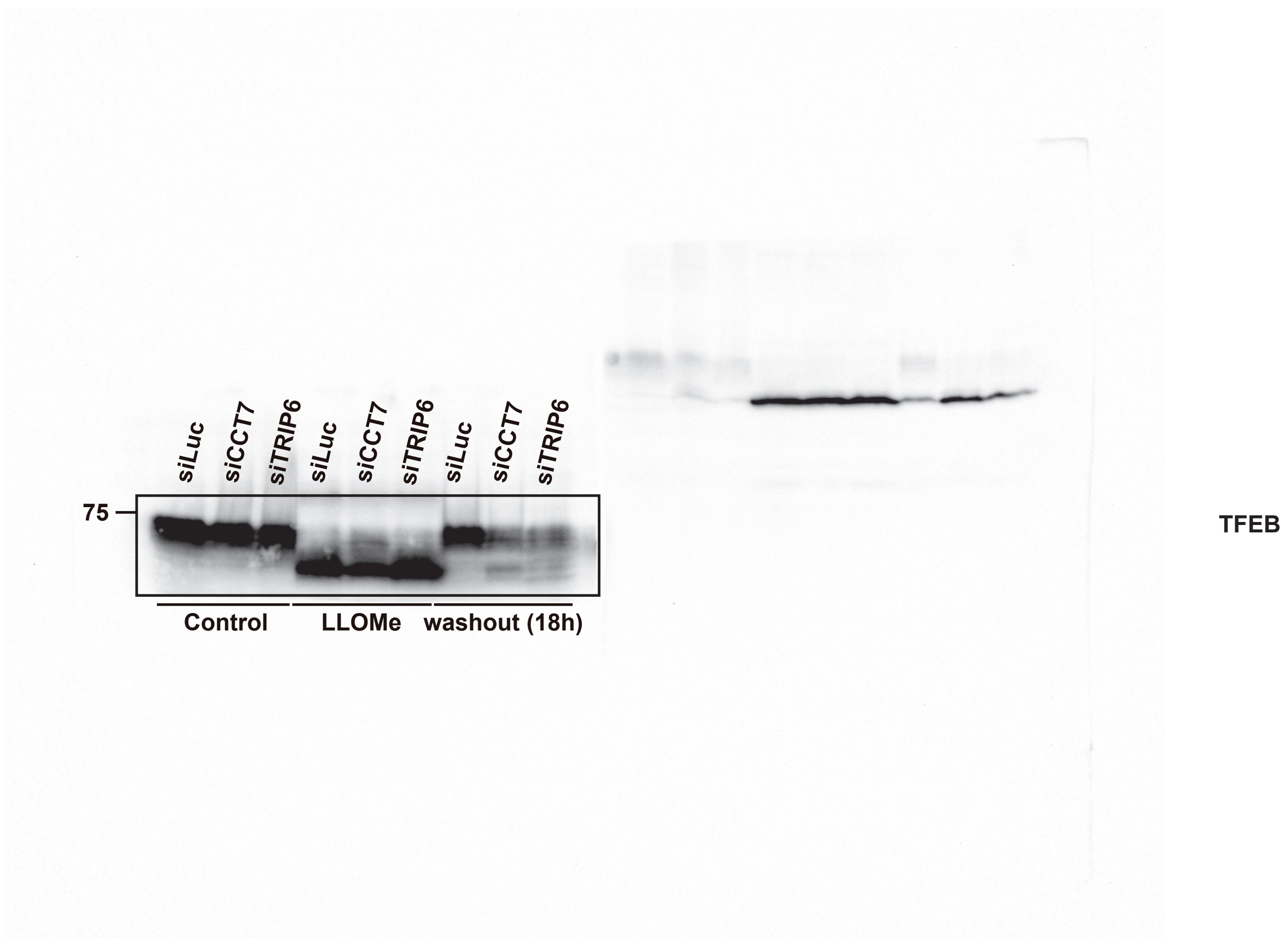

siLuc
siCCT7
siTRIP6
siLuc
siCCT7
siTRIP6
siLuc
siCCT7
siTRIP6
75
Control
LLOMe
washout (18h)
TFEB

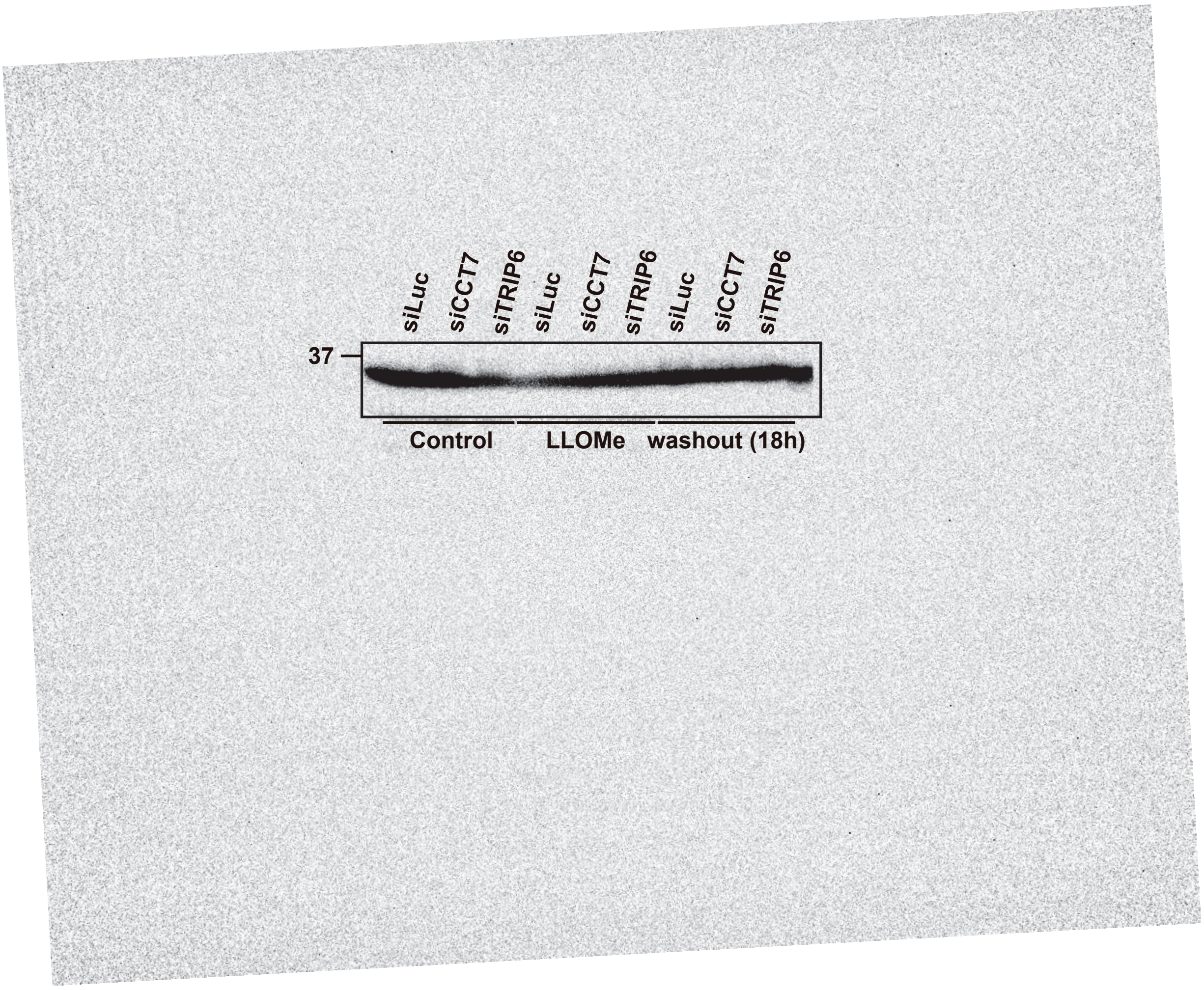
siLuc
siCCT7
siTRIP6
siLuc
siCCT7
siTRIP6
siLuc
siCCT7
siTRIP6
37
Control
LLOMe
washout (18h)

GAPDH

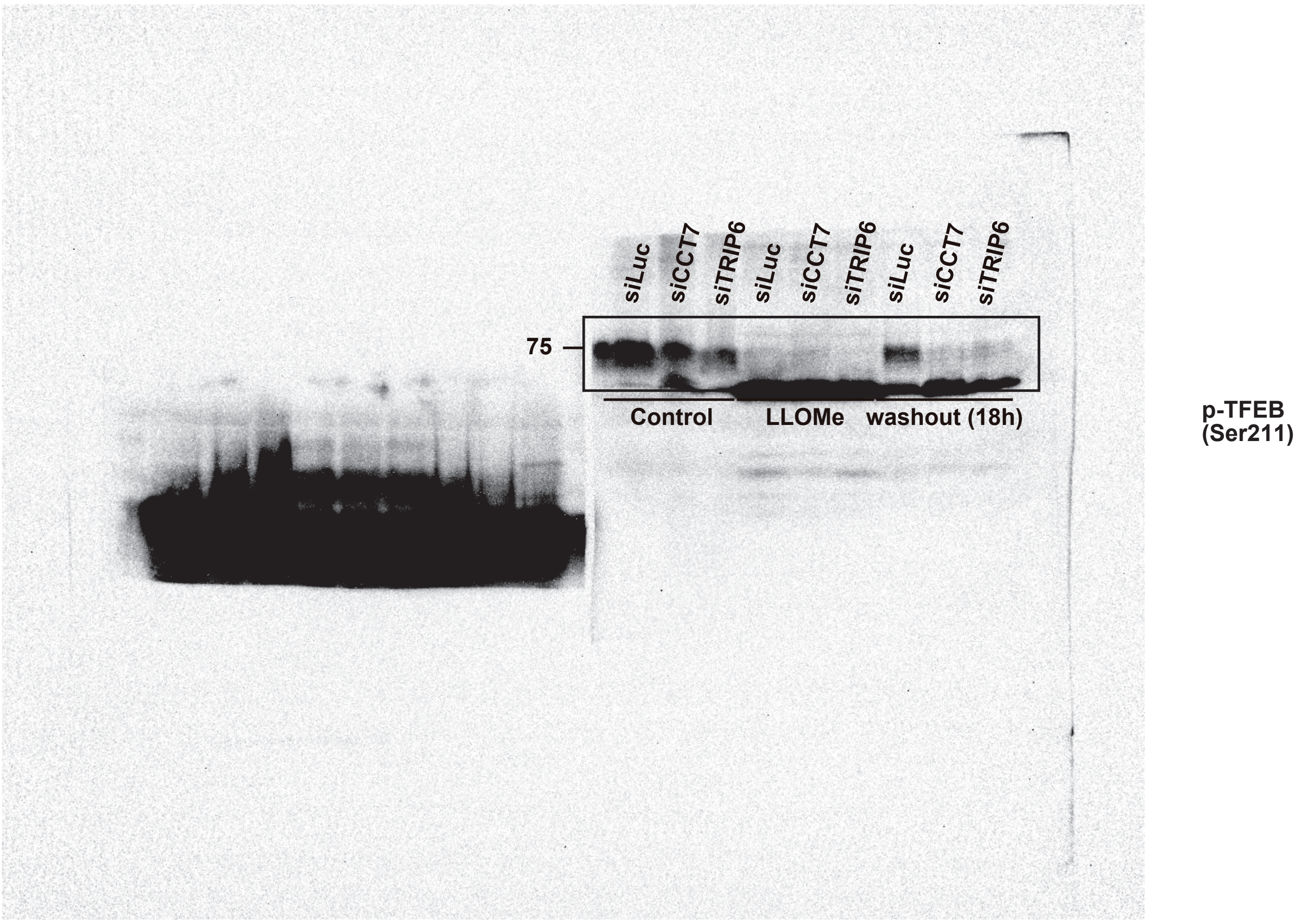
siLuc
siCCT7
siTRIP6
siLuc
siCCT7
siTRIP6
siLuc
siCCT7
siTRIP6
75
Control
LLOMe
washout (18h)
p-TFEB
(Ser211)

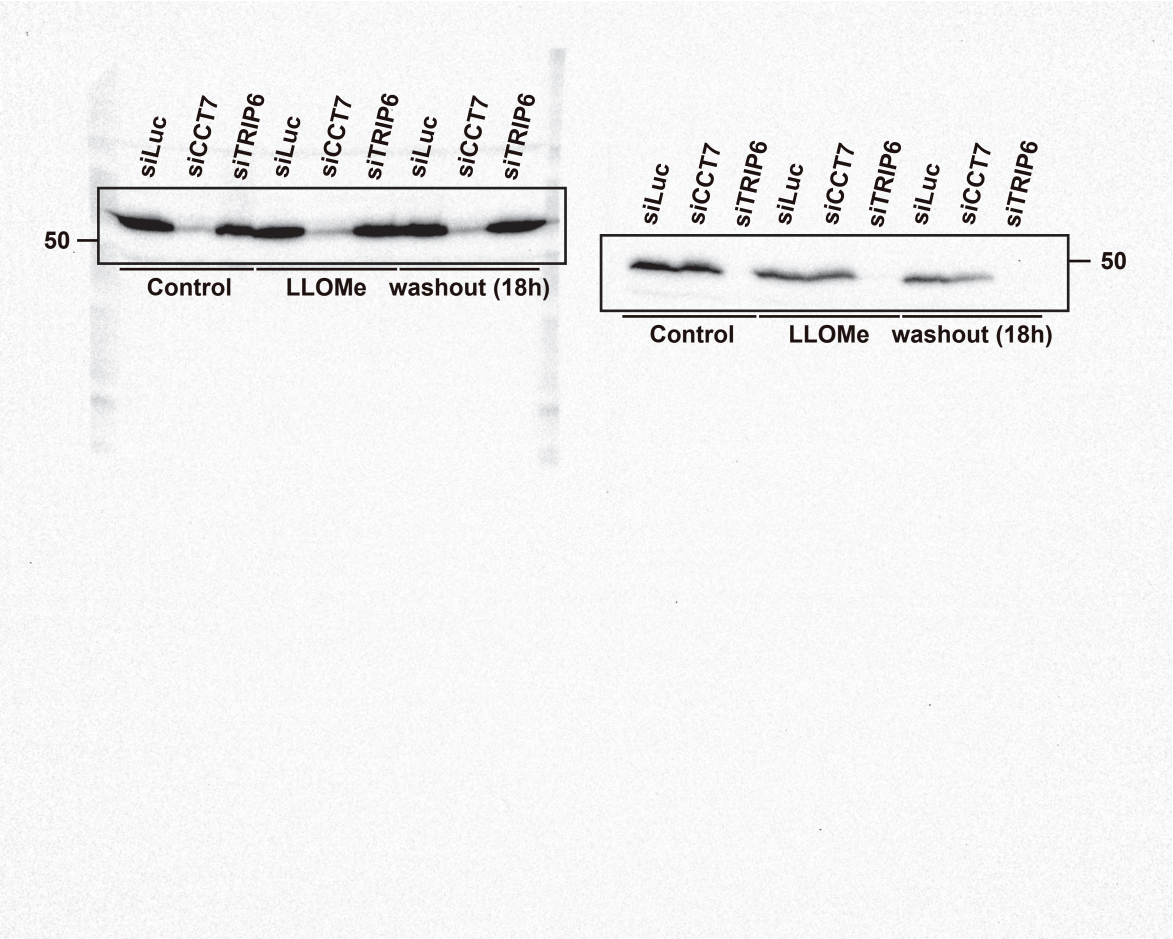
siLuc
siCCT7
siTRIP6
siLuc
siCCT7
siTRIP6
siLuc
siCCT7
siTRIP6
50
Control
LLOMe
washout (18h)
siLuc
siCCT7
siTRIP6
siLuc
siCCT7
siTRIP6
siLuc
siCCT7
siTRIP6
50
Control
LLOMe
washout (18h)

CCT7 TRIP6
